# Annotations

**Published:** 1894-12-29

**Authors:** 


### ANNOTATIONS.

### Sane Persons in Private Asylums.

Many good people honestly believe that sane persons are sometimes locked up in private lunatic asylums, the victims of cruel conspiracies among interested persons. Of this class was the late Charles Reade, the novelist. It is very desirable that suspicions of this kind should be allayed; and the *British Medical Journal* publishes some facts which are of interest in this connection, and which also have a decidedly amusing side. Reade was the president of a club which had for its principal object the deliverance of sane victims from mad doctors. On one occasion Mr. Ernest Hart was invited to a club dinner, in order that he might see for himself how very sane the members of the club were, and how very much in earnest also they could be in their efforts to release their sane brethren whom they imagined to be so wickedly incarcerated in private asylums. At the close of the dinner the toasts were proposed, or rather the toast; for, as Charles Reade explained, only one toast was necessary in the case of an assembly of so serious a kind. That toast was, of course, "The Queen." No sooner was it proposed than a tall man at the bottom of the table rose up, and with the utmost gravity said: "I thank you for the honour you have done me; I am the Queen." When he had been promptly pulled down, kicking and struggling all the while, another gentleman rose up protesting that the tall man could not be the Queen, since he himself was the King. Needless to say, Mr. Reade left the room in disgust; and it is not recorded that he presided at any more dinners of the kind. We do not say that all those who hold briefs against private lunatic asylums are themselves lunatics, but it is clearly more than probable that some of them may be.

### The Clinical Society.—Discussion on Antitoxin.

The discussion at the Clinical Society resolved itself chiefly into a relation of their experiences by various members and visitors who had made use of antitoxin in the treatment of diphtheria, and among them there seemed a general consensus in favour of the remedy. An attempt which was made to draw from the readers of the paper a more definite limitation of the cases in which its use was to be specially recommended, and of those in which its application was to be looked upon as useless, failed to lead to any greater exactitude of knowledge, and it would seem that at present we must be content with the proof which has been offered—statistical and other—that in some ill understood way the remedy does good, and that if it is to do its greatest good it must be given at the earliest possible period in the disease. Dr. Newman, of Glasgow, drew attention to the fact, which has already been pointed out in our columns, that when serum was injected a good deal of stuff besides antitoxin was being used, which had no remedial efficacy, and that probably something yet might be done in the direction of a further isolation of the antitoxic material. He gave some account of cases which had been under his care showing that the results had been so far satisfactory. A speaker drew attention to the number of different preparations of antitoxin which were at present "in the market," and laid emphasis on the importance of every observer recording not only the dose, but the source of the remedy. Professor Sims Woodhead pointed out that in diphtheria two very different toxins were produced—one which was formed locally at the seat of the disease by the bacteria themselves, and another by the reaction of this upon the tissues—and there was some probability that the neutralising effect of the serum of immunised animals was principally exerted upon the first of the two; the apparent delay in its action being possibly due to the time occupied in eliminating the other form. Dr. McCombie, of the South-Eastern Hospital, had had thirty-one cases, of which three had died and one more would in all probability do so; but he drew attention to the weakness of statistics when derived only from small numbers spread over short periods, for, taking the mortality at his hospital month by month, it had varied between 42 per cent. and 18 per cent. Curiously enough, the month which showed the lowest death-rate immediately followed one which had nearly the highest, viz., 37 per cent.; so that if on August 1st he had begun to use antitoxin, he might have been entirely deceived as to the result, seeing that, without its use at all, the mortality had fallen in that month to less than half what it was in July.

### The Electrolytic Treatment of Pruritus.

Practical suggestions for the treatment of so troublesome and annoying an affection as pruritus are always welcome. Dr. H. Leloir, Professor of Dermatology at the University of Lille, in the course of a paper on "Dermato-neuroses and their Treatment" in the *British Journal of Dermatology*, gives his experience of the treatment of various forms of pruritus by electrolysis. "I have cured," he says, "many cases of cutaneous pruritis, of vulvar and of anal pruritus, which had been absolutely rebellious to all previous treatment." The method, he reports, is painful, and is only applicable to cases of pruritus which are strictly localised. The eczematous or lichenoid conditions secondary to the pruritus disappeared as well as the itching. In some cases the treatment entirely failed. The process employed is that of connecting the patient with one pole of a powerful static machine, placed on a small table with glass legs. A metallic point connected with the other pole is then held at a distance of about 0·10 to 0·15 cent. from the affected area. There are some little deficiencies in the report. It is not stated, for example, how many applications are required; nor is it indicated whether or not the cure is permanent or of long duration. No attempt is made to explain the physiological *modus operandi*. The treatment, at any rate until it is more philosophically explained, is therefore to be considered as a mere additional clinical resource of a purely empirical character, to be employed in case of urgent need. The probability would seem to be that the sensitiveness of the nerves involved is merely temporarily exhausted; and if that be so, the "cure" is hardly worth calling a "cure." The real cure of pruritus, as of many other dermato-neuroses, is the restoration of such a degree of health and vigour to the nerve centres, and such an improvement of the blood circulation, as will bring about a generally normal physiological condition throughout the whole body. When that is done, the local pruritus will cease of itself.

